# Saos‐2 cells cultured under hypoxia rapidly differentiate to an osteocyte‐like stage and support intracellular infection by *Staphylococcus aureus*

**DOI:** 10.14814/phy2.15851

**Published:** 2023-11-06

**Authors:** Anja R. Zelmer, Yolandi Starczak, Lucian B. Solomon, Katharina Richter, Dongqing Yang, Gerald J. Atkins

**Affiliations:** ^1^ Biomedical Orthopaedic Research Group, Centre for Orthopaedic & Trauma Research, Faculty of Health and Medical Sciences University of Adelaide Adelaide South Australia Australia; ^2^ Centre for Orthopaedic & Trauma Research, Faculty of Health and Medical Sciences University of Adelaide Adelaide South Australia Australia; ^3^ Department of Orthopaedics and Trauma Royal Adelaide Hospital Adelaide South Australia Australia; ^4^ Richter Lab, Department of Surgery Basil Hetzel Institute for Translational Health Research, University of Adelaide Adelaide South Australia Australia

## Abstract

The intracellular infection of osteocytes represents a clinically important aspect of osteomyelitis. However, few human osteocyte in vitro models exist and the differentiation of immature osteoblasts to an osteocyte stage typically takes at least 4‐weeks of culture, making the study of this process challenging and time consuming. The osteosarcoma cell line Saos‐2 has proved to be a useful model of human osteoblast to mature osteocyte differentiation. Culture under osteogenic conditions in a standard normoxic (21% O_2_) atmosphere results in reproducible mineralization and acquisition of mature osteocyte markers over the expected 28–35 day culture period. In order to expedite experimental assays, we tested whether reducing available oxygen to mimic concentrations experienced by osteocytes in vivo would increase the rate of differentiation. Cells cultured under 1% O_2_ exhibited maximal mineral deposition by 14 days. Early (*COLA1*, *MEPE*) and mature (*PHEX*, *DMP1*, *GJA1*, *SOST*) osteocyte markers were upregulated earlier under hypoxia compared to normoxia. Cells differentiated under 1% O_2_ for 14 days displayed a similar ability to internalize *Staphylococcus aureus* as day 28 cells grown under normoxic conditions. Thus, low oxygen accelerates Saos‐2 osteocyte differentiation, resulting in a useful human osteocyte‐like cell model within 14 days.

## Graphical abstract

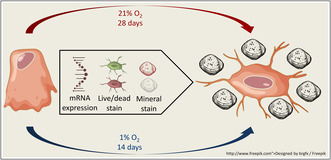

## 1 INTRODUCTION

Osteocytes are the major and most long‐lived bone cell population, potentially living for decades in vivo (Prideaux et al., 2016). Osteocytes are critical for bone health and play numerous physiologic roles. The bone is a generally hypoxic tissue with oxygen levels in the bone marrow reported to vary between 1%–6% O_2_ (Mohyeldin et al., 2010), thus osteocytes physiologically experience hypoxia in vivo. Although several pathways have been identified to mediate adaptation to hypoxia, the primary mediators are the hypoxia‐inducible factors (HIFs). Hypoxia‐inducible factor‐1 alpha (HIF‐1α) is the principal transcription factor mediating adaptive responses to reduced O_2_ levels. HIF‐1α protein levels are naturally regulated by proteosomal degradation following prolyl‐hydroxylation by a family of prolyl‐hydroxylases (PHDs1‐3) (Barrett et al., 2011). During hypoxia, prolyl‐hydroxylation is blocked, leading to HIF‐1α accumulation, nuclear translocation, and dimerization with HIF‐1β, initiating HIF‐responsive gene transcription by binding to hypoxia‐responsive elements (HREs) in target gene promoters. Hypoxia signaling is an important regulator of normal bone mass, demonstrated by young *Hif1a* ^*null*^ mice having reduced cortical bone volume, which is reversed in aged mice (Riddle et al., 2011). Activation of the HIF1α pathway has been reported to promote bone modeling in long bones (Wang et al., 2007). It has also been suggested that HIF1α inhibits Wnt signaling by activating SOST expression in osteoblasts, hence halting osteogenesis (Chen et al., 2013).

Recent findings suggest that osteocytes play an important role in chronic osteomyelitis, since bacteria can persist intracellularly in these cells and potentially spread throughout the lacunocanalicular system (de Mesy Bentley et al., 2018; Yang et al., 2018). The human osteosarcoma cell line Saos‐2 (or HTB‐85™), long known to mineralize its extracellular matrix, has been shown to provide a model for intracellular infections as an osteoblast and osteocyte‐like host cell (Gunn et al., 2021). We previously reported the ability of Saos‐2 cells to differentiate to an osteocyte‐like stage by 28 days (28 days) of osteogenic culture (Prideaux et al., 2014). The transition from an osteoblast‐like to an osteocyte‐like phenotype can be monitored by the acquisition of mature osteocyte marker expression concomitant with mineral deposition. We recently showed the utility of these 28 days cultures for the study of intra‐osteocytic infection with *Staphylococcus aureus* (Gunn et al., 2021). However, 28 days of pre‐culture prior to performing experimentation presents a logistical barrier and is costly in terms of both tissue culture reagents and time. We hypothesized that culture of these cells under low oxygen conditions, which is physiologically more representative of bone than standard culture in atmospheric oxygen, would promote their differentiation. In this study, we therefore compared osteogenic differentiation under atmospheric oxygen (21%) and a nominal hypoxic concentration of 1% O_2_.

## 2 MATERIALS AND METHODS

### 2.1 Cell culture

Saos‐2 cells (HTB‐85, cells from an 11 year old female patient; ATCC, Australia) were maintained in growth media consisting of αMEM (Gibco) supplemented with 10% v/v fetal calf serum (FCS) and standard tissue culture additives (10 mM HEPES, 2 mM L‐Glutamine, penicillin/streptomycin each 1 unit/mL (Thermo Fisher)) at 37°C/5% CO_2_ (Gunn et al., 2021; Prideaux et al., 2014; Yang et al., 2018). Cells were confirmed to be mycoplasma‐free using MycoStrip™ (Invivogen).

For experimentation, cells were seeded at a density of 1 × 10^4^ cells/well in Nunclon Delta Surface (Thermo Scientific) 48 well plates or 2 × 10^4^ cells/well in 24 well plates (Thermo Scientific) and maintained with bi‐weekly media change. To achieve an osteocyte‐like phenotype, Saos‐2 cells were switched to differentiation media at confluence, consisting of αMEM supplemented with 5% v/v FCS, standard tissue culture additives plus 50 μg/mL ascorbate 2‐phosphate and 1.8 mM potassium di‐hydrogen phosphate (AJAY Chemicals) and then cultured either at 37°C/5% CO_2_ for 28 days in a standard atmosphere tissue culture incubator (Heracell Vios 160i, Thermo Fisher) (Prideaux et al., 2014) or at 37°C/5% CO_2_/1% O_2_ in a nitrogen‐controlled hypoxic incubator (New Brunswick Galaxy 170R, Eppendorf). Prior to media changes, medium for hypoxia cultures was equilibrated under 1% O_2_ to reduce dissolved oxygen levels. All experiments were performed in at least biological quadruplicates.

### 2.2 Measurement of cell viability

Cells were cultured, as described above, on Cell Imaging Plates (24 well, Cat. No. 0030741005, Eppendorf). After 7‐, 14‐, 21‐, and 28‐days in differentiation media, the cells were incubated for 5 min with eBioscience™ Calcein Violet 450 am Viability Dye (Cat. No. 65–0854‐39, Invitrogen) and Ethidium Homodimer III (Cat. No. 40051, Biotium). Confocal images were taken with an Olympus FV3000 confocal microscope (Olympus) and processed with Fiji ImageJ to obtain the relative intensity.

### 2.3 Scanning electron microscope (SEM)

To visualize cells by SEM, cells were cultured as described above on glass slides in a 24‐well plate for 14 days and processed, as described previously (Kumarasinghe et al., 2012). The cells were washed with PBS and fixed with 1.25% v/v glutaraldehyde, 4% w/v sucrose, and 4% w/v paraformaldehyde in PBS for 24 h. Samples where then dehydrated with an increasing ethanol concentration series (70%, 60%, 95%, 100%) of 0.5 mL for 5 min each, air dried and carbon coated (Denton Vacuum DV‐502), and examined on an electron microscope (Hitachi SU7000 SEM, Hitachi‐Hightech of Japan).

### 2.4 Measurement of in vitro mineralization

Cells were harvested 7‐, 14‐, 21‐, and 28‐days after changing to differentiation media. They were rinsed with phosphate buffered saline (PBS, Cat. No. D1408, Sigma‐Aldrich), fixed with 10% formalin for 10 min, rinsed three times with milli‐Q water (MQ). Four replicates were stained for calcium with 2% (in demineralized water, pH adjusted to 4.1–4.3 using 0.5% ammonium hydroxide) Alizarin Red (Cat. No. A5533, Sigma‐Aldrich) for 5 min and rinsed with water until the wash was clear. Cells were imaged and then processed for quantitative analysis (Prideaux et al., 2014). For this, cells were incubated for 30 min with 10% acetic acid on a shaker and then transferred into 1.5 mL reaction tubes. After mixing the lysate was heated for 10 min at 85°C and then cooled on ice for 5 min. The samples were then centrifuged at 20,000*g* for 15 min and the supernatant transferred to fresh tubes. The pH was adjusted to 4.1–4.5 with 10% ammonium hydroxide. Samples were read at OD_405 nm_ and quantified using a standard curve. Four replicates were stained for deposited phosphate using the von Kossa stain (Atkins et al., 2009). Briefly, cells were incubated with 1% AgNO_3_ (Sigma‐Aldrich) for 30 min under light exposure, rinsed three times with MQ and incubated with Na_2_S_2_O_3_ (Sigma‐Aldrich) for 5 min, rinsed three times with MQ and then imaged.

### 2.5 Measurement of alkaline phosphatase activity

Cells were harvested 7‐, 14‐, 21‐, and 28‐days after changing to differentiation media. They were rinsed with PBS, fixed with 10% buffered formalin for 10 min, then rinsed three times with MQ. Four replicates were stained for alkaline phosphatase (ALP) with StayRed (AB103741, Abcam) according to the manufacturer's instructions. ALP staining was quantified by image analysis of at least four regions of interest per well, both stained and unstained. Images were converted to RGB and the red regions converted to black and white with a standardized threshold and measured with Fiji ImageJ to obtain the relative intensity.

### 2.6 Measurement of gene expression by real‐time RT‐PCR

For the quantification of gene expression of Collagen Type I (*COLA1*), Runt‐related transcription factor 2 (*RUNX2*), bone GLA‐containing protein‐1 (*BGLAP*), Connexin 43 (*GJA1*), Hypoxia‐inducible factor 1‐alpha (*HIF1A*), matrix extracellular phosphoglycoprotein (*MEPE*), phosphate‐regulating neutral endopeptidase (*PHEX*), dentin matrix protein 1 (*DMP1*) and sclerostin (*SOST*), total RNA was isolated using TRI reagent (Cat No. T9424, Sigma‐Aldrich), and complementary DNA (cDNA) templates were prepared using the iScript gDNA Clear cDNA Synthesis kit (Cat. No. 1725035, BioRad), as per manufacturer's instructions. Real‐time RT‐PCR reactions were then performed in technical duplicates to determine the mRNA levels of target genes using RT2 Forget‐Me‐Not™ EvaGreen qPCR Mastermix (Cat. No. 31041, Biotium, Assay Matrix) on a CFX Connect Real‐Time PCR System (BioRad). The sequences of the oligonucleotide primer sets targeting each gene are listed in Table 1. At least one primer per pair was designed to flank an intron‐exon boundary in order to be mRNA‐specific; custom primers were purchased from Sigma‐Aldrich. Gene expression relative to the level of *ACTB* mRNA was calculated using the 2^−∆Ct^ method.

**TABLE 1:** Oligonucleotide primer sequences used for qPCR.

Gene (reference)	Forward primer (5′‐3′)	Reverse primer (5′‐3′)
*ACTB* (Alshabrawy et al., 2022)	AAGAGATGGCCACGGCT	CAATGATCTTGATCTTCATTGTGC
*BGLAP* (Atkins et al., 2007)	ATGAGAGCCCTCACACTCCTCG	GTCAGCCAACTCGTCACAGTCC
*COL1A1*	AGTGTGGCCCAGAAGAACTG	CCGCCATACTCGAACTGGAA
*DMP1* (Ormsby et al., 2016)	GATCAGCATCCTGCTCATGTT	AGCCAAATGACCCTTCCATTC
*GJA1* (Yang et al., 2015)	AAGTGAAAGAGAGGTGCCCA	GTGGAGTAGGCTTGGACCTT
*HIF1A*	GTGTGAATTACGTTGTGAGTGGT	AACCGGTTTAAGGACACATTCT
*MEPE* (Ormsby et al., 2021)	AGATTCTCAAAGATGCGAGTTTTC	CCTCTGCTCTTCCACACAGC
*PHEX* (Ormsby et al., 2021)	CATCCAATGAACATATCTTGAAGC	ACTTGTAAAGGGCATCCCGATAA
*RUNX2*	CCTTCAAGGTGGTAGCCCCT	GGTGAAACTCTTGCCTCGTC
*SOST* (Ormsby et al., 2016)	GCTGTACTCGGACACGTCTT	ACCGGAGCTGGAGAACAACA

### 2.7 Bacterial culture and infection assay

Infection assays were performed essentially as previously described (Gunn et al., 2021; Yang et al., 2018). Two clinical isolates from patients undergoing digital amputation for osteomyelitic diabetic foot infection (designated strains B and W) of *S. aureus* were grown until log‐phase in Terrific Broth (TB; Thermo Fisher) on a 37°C/ 200 rpm rocking platform, pelleted at 20,000×*g* for 5 min, and resuspended in PBS. The viable bacterial cell concentration for each experiment was estimated from a colony forming unit (CFU)/ml versus OD_630nm_ standard curve, then validated by plating dilutions on Nutrient Broth (NB; 10 g/L Peptone +5 g/L sodium chloride +3 g/L beef extract; Chemsupply)‐agar, consisting of NB with 1.5% w/v bacteriological agar (Sigma‐Aldrich). The required number of bacteria for an estimated multiplicity of infection (MOI) of 0.5 was diluted into PBS and validated by plating dilutions on NB agar and recording CFU number. Differentiated Saos‐2 cells were infected for 2 h and extracellular bacteria cleared with 1% w/v Lysostaphin (Sigma‐Aldrich) in differentiation medium. Cultures were incubated either at 37°C/5% CO_2_ in normoxia or at 37°C/5% CO_2_/1% O_2_, as described above. The cells were harvested 24 h post‐infection and lysed in sterile water for CFU analysis, as above.

### 2.8 Statistical analysis

Two‐way ANOVA with Tukey's post‐hoc tests were used to compare differences during the time course of Saos‐2 differentiation. To compare specific pairs, unpaired *T*‐tests were used. All analysis was performed using GraphPad Prism 9 software (GraphPad). Values for *p* < 0.05 were considered significant. Plotted data are shown as means ± standard deviation. All experiments were performed in at least biological quadruplicates.

## 3 RESULTS

### 3.1 Effects of oxygen level on cell viability

**FIGURE 1:**
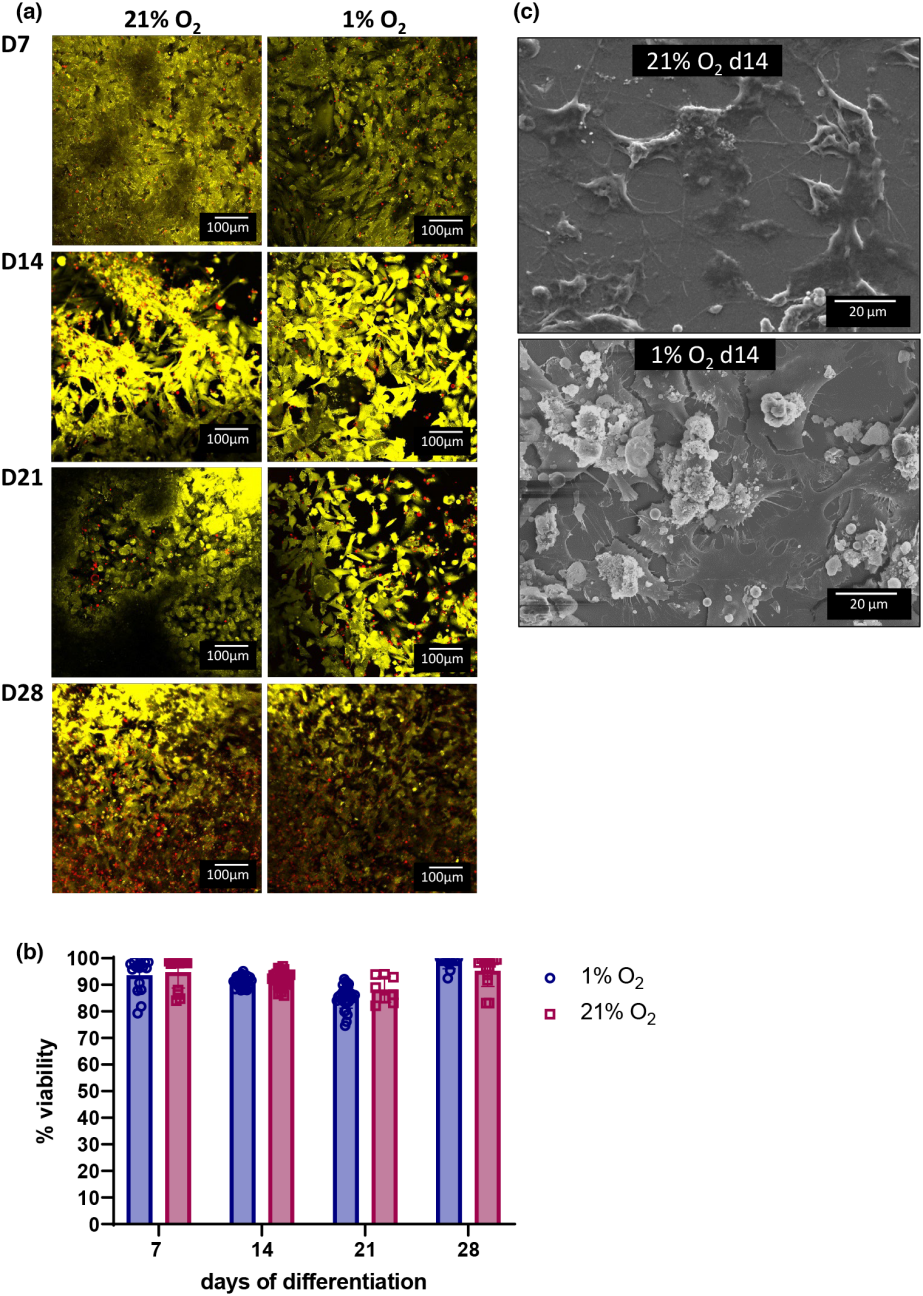
Hypoxia maintains Saos‐2 cell growth and viability. Saos‐2 cells cultured under 1% or 21% O_2_ under osteogenic conditions were assayed for cell viability at weekly intervals up to 28‐days and stained with Calcein Violet 450 am Viability Dye (*green*) and Ethidium Homodimer III (*red*) for live/dead confocal imaging. (a) Representative images; (b) quantified percentage of live stain to dead stain. For each time point and condition, four wells were imaged at least twice; (c) SEM analysis of Saos‐2 morphology after differentiation for 14 days under either 1% or 21% O_2._

At all time points examined, cells under either normoxic or 1% O_2_ culture condition appeared uniformly viable by phase contrast microscopy and by Calcein Violet and Ethidium Homodimer III staining (Figure 1a). Live/dead staining confirmed that the proportion of dead cells did not significantly change during the differentiation, nor was it different under either culture condition, with at least 85% viability throughout (Figure 1b), indicating that Saos‐2 cells can survive under 1% O_2_ levels, as well as under normoxia for at least 28 days. Morphological analysis by SEM revealed the presence of dendritic processes at day 14 under either oxygen condition (Figure 1c).

### 3.2 Effects on in vitro mineralization

**FIGURE 2:**
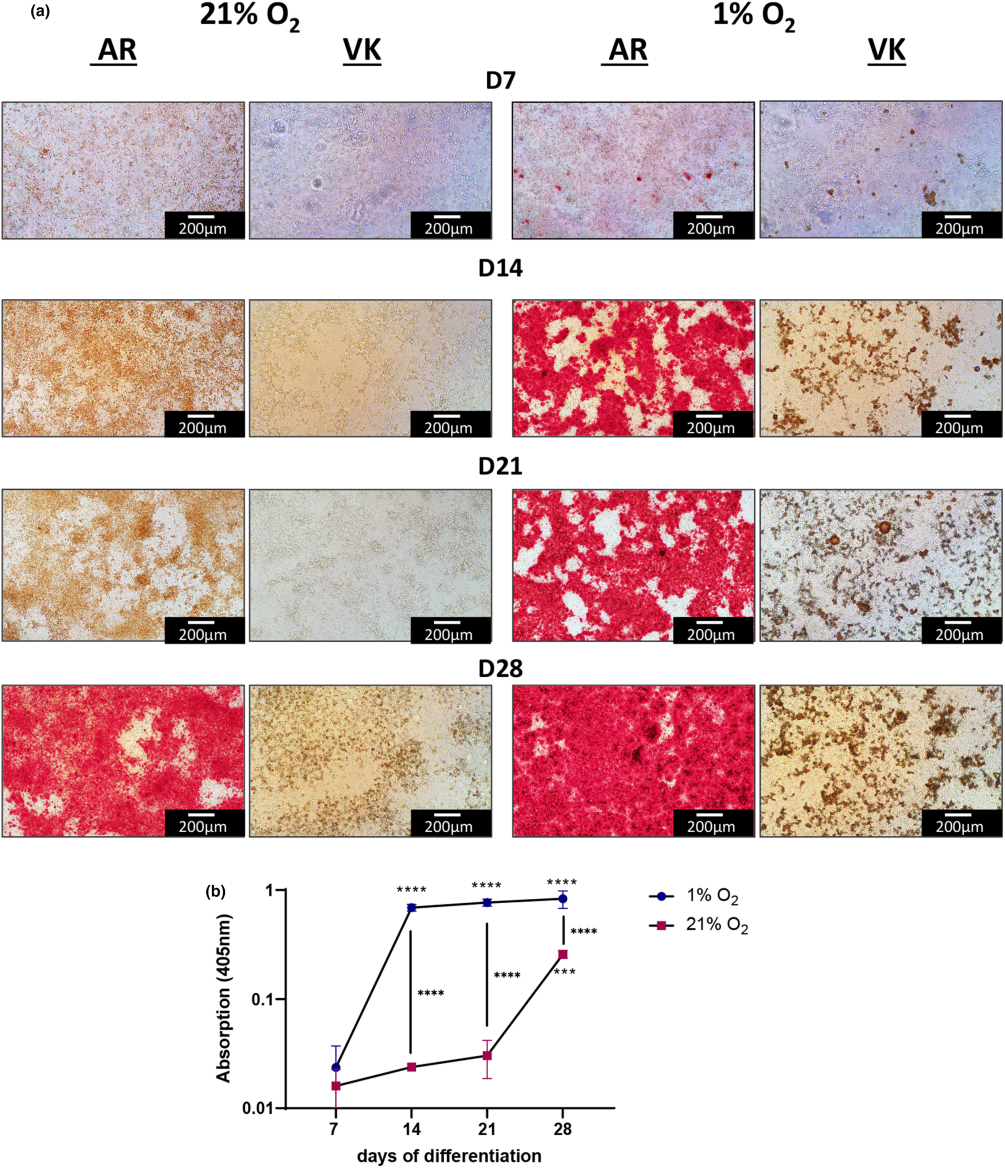
Hypoxia accelerates Saos‐2 in vitro mineralization. (a) Saos‐2 cells cultured under 21% O_2_ or 1% under osteogenic conditions were assayed at weekly intervals up to 28‐days for calcium deposition using Alizarin Red (AR) and phosphate deposition using von Kossa (VK) stain. Experiments were conducted twice with quadruplicate wells and at least three regions of each well were imaged. (b) Alizarin Red quantification at 405 nm absorption. Asterisks above or below a time point indicate significant difference to day 7: ***p < 0.001, ****p < 0.0001. Asterisks between curves indicate significant difference between conditions: ****p < 0.0001. Four wells per time point and condition were used for quantification.

Saos‐2 cultures differentiated under 1% O_2_ mineralized more rapidly than under 21% O_2_, as determined by Alizarin Red staining for calcium and von Kossa staining for phosphate (Figure 2). Cells under 1% O_2_ reached a significantly higher mineralization level at 14 days than the cells under 21% O_2_ did after 28 days and was maximal at the 14 day time point (Figure 2b).

**FIGURE 3:**
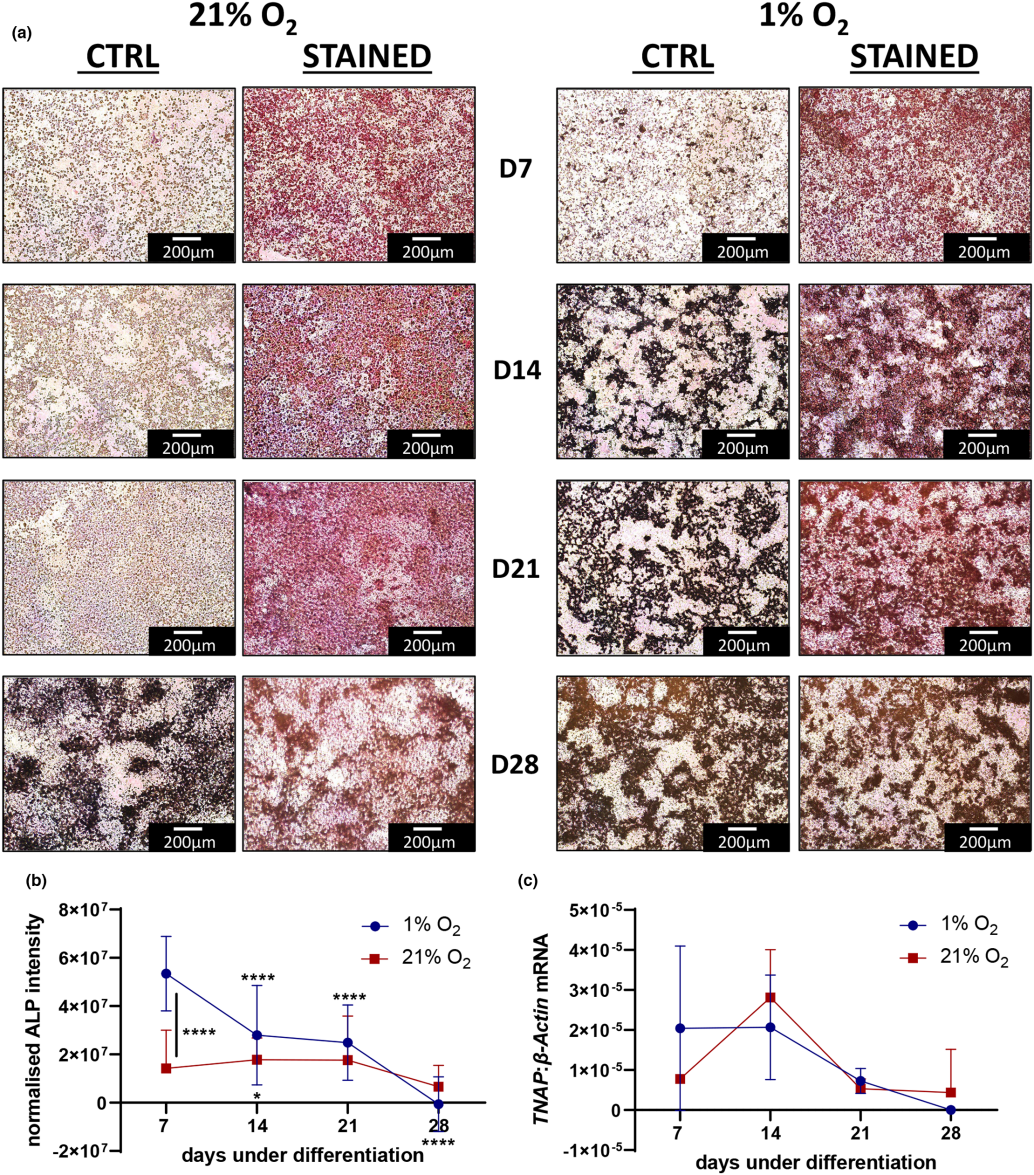
Hypoxia increases alkaline phosphatase activity. (a) Saos‐2 cells after 7‐, 14‐, 21‐ and 28‐days of differentiation under 1% or 21% O_2_ stained with StayRed (Abcam) to measure alkaline phosphatase activity. Each condition was performed in quadruplicate and at six regions of each well were imaged. (b) Quantification of StayRed Stain normalized to unstained control with 1% (blue circle) or 21% O_2_ (red square). Asterisks above a time point indicate significant difference to the corresponding day 7 value: ***p < 0.001, ****p < 0.0001. (c) Relative *TNAP* mRNA expression after 7‐, 14‐, 21‐, and 28‐days of differentiation under 1% (blue circle) or 21% O_2_ (red square).

Consistent with the above, under 1% O_2_ increased ALP activity was detectable at an earlier time point than under 21% O_2_ (Figure 3a,b). This relationship was reflected by a similar trend in *TNAP* mRNA expression (Figure 3c).

### 3.3 Effects on gene expression

**FIGURE 4:**
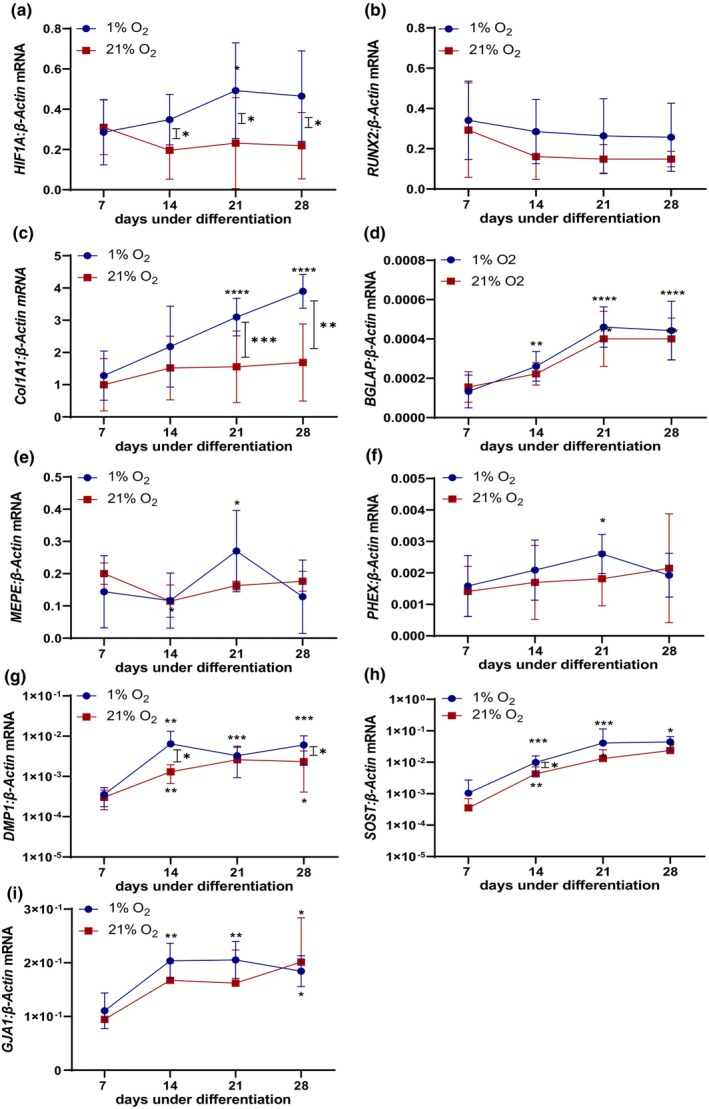
Effects of differentiation under hypoxia on gene expression. Saos‐2 cells cultured under 1% or 21% O_2_ under osteogenic conditions were assayed at weekly intervals up to 28‐days for gene expression by RT‐PCR for (a) *HIF1A*, (b) *RUNX2*, (c) *COL1A1*, (d) *BGLAP*, (e) *MEPE*, (f) *PHEX*, (g) *DMP1*, (h) *SOST*, and (i) *GJA1*. Data are normalized to the mRNA expression of β‐Actin (*ACTB*). Significant differences between culture conditions are depicted by intervening asterisks; significant differences to the corresponding day 7 value are represented by asterisks above (1% O_2_) or below (21% O_2_) each curve; *p < 0.05, **p < 0.01, ***p < 0.001, ****p < 0.0001. The experiment was conducted with six biological replicates. Each qPCR reaction was performed in technical duplicate.

There was a significant higher expression of *HIF1A* mRNA under 1% O_2_ compared to 21% O_2_, however only from day 14 of culture (Figure 4a), consistent with the cells responding metabolically to the low oxygen conditions. Expression of the major osteoblastic transcription factor *RUNX2* was elevated from day 14 under 1% O_2_, (Figure 4b) consistent with hypoxia having an osteogenic effect on the cells (Komori, 2019). The expression of *COL1A1* mRNA increased under both culture conditions, as expected, but to a greater extent under 1% O_2_ (Figure 4c), consistent with increased collagen type I bone matrix production and the increased mineralization under hypoxic conditions. The expression of late osteoblast/osteocyte markers *BGLAP*, *MEPE*, and *PHEX* (Figure 4d–f) followed a similar pattern under both culture conditions, and at 21 days were significantly elevated under 1% O_2_ in the case of *MEPE* and *PHEX*, consistent with enhancement of osteogenic differentiation under hypoxic conditions. Elevated mRNA expression of the mature osteocyte markers *DMP1*, *SOST*, and *GJA1* was observed as expected (Boukhechba et al., 2009; Poole et al., 2005; Prideaux et al., 2014; Sawa et al., 2019; Toyosawa et al., 2001) but these were both relatively increased and plateaued under 1% O_2_ by day 14 (Figure 4g–i), indicating more rapid acquisition of an osteocyte‐like phenotype (within 14 days) than under normoxic conditions.

### 3.4 Effects on support of intracellular bacterial infection

**FIGURE 5:**
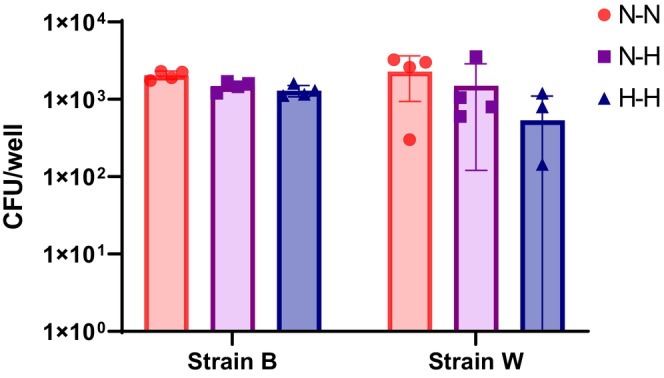
Effects of differentiation under hypoxia on osteocyte support of intracellular infection. Cells differentiated under 1% O_2_ (H) for 14 days or 21% O_2_ (N) for 28 days were exposed to two strains of *S. aureus* (B and W) at MOI 0.5, as described in Materials and Methods, then cultured under either H or N conditions, creating three conditions (N‐N, N‐H, and H‐H). After a further 24 h, CFU counts were performed on cell lysates, as described. Experiments were conducted in biological quadruplicate. No significant differences were recorded.

Having established that 14 days was sufficient to generate an osteocyte‐like phenotype under 1% O_2_, we next tested the suitability of these cells to study osteocytic intracellular infection with *S. aureus*. Differentiated Saos‐2 cells cultured under either normoxia (N; 21% O_2_) for 28 days or hypoxia (H; 1% O_2_) for 14 days were infected with an identical MOI of two clinical osteomyelitis‐associated isolates of *S. aureus* and then each cultured overnight in either oxygen concentration, generating 3 culture conditions: N‐N, N‐H, and H‐H. For all culture combinations, there was less than 0.5‐log difference in the degree of cellular invasion (Figure 5), indicating that the day 14 hypoxia differentiated osteocyte‐like cells faithfully replicated the infection dynamics of the published day 28 normoxia model (Gunn et al., 2021).

## 4 DISCUSSION

Saos‐2 cells are the only widely validated human transformed osteoblastic cell line demonstrated to differentiate to an osteocyte‐like stage (Prideaux et al., 2014). To date, one major limitation of such differentiation models is the long period of time, typically 28–35 days, required before the cells are ready to test as an osteocyte‐like cell; here, we showed that differentiation under 1% oxygen reduces the differentiation period to 14 days, permitting more timely execution of these experiments.

Culture under 1% O_2_ induced higher expression of *HIF1A* mRNA compared to 21% O_2_ from day 14 of culture, consistent with the cells responding metabolically to the reduced available oxygen. Interestingly, there was no change in *HIF1A* levels at the day 7 time point under 1% O_2_. It is possible that because oxygen levels were not controlled during media changes beyond prior equilibration at 1% O_2_ that the hypoxic effect was slower to manifest or that the early undifferentiated cells were naturally adapted to a low oxygen environment; in either case, it is also possible that the extensive mineralization by day 14 further reduced oxygen availability to the cells and this was an additional trigger for *HIF1A* upregulation. Regardless, the accelerated differentiation process under 1% O_2_ was reflected in more rapid in vitro mineralization, indicated by maximal Alizarin Red staining by day 14 as a measure of calcium incorporation, increased ALP activity at early time points, permissive for phosphate incorporation, and elevated *COL1A1* mRNA expression as a measure of earlier collagen type 1 organic matrix production.

*RUNX2* expression by immature osteoblasts is fundamentally permissive for their osteogenic differentiation (Komori, 2010, 2019), thus the elevated expression of *RUNX2* mRNA under 1% O_2_ compared to standard normoxic conditions is strongly supportive of an osteogenic response. This was reflected in the associated increase in expression of all osteogenic genes examined. *PHEX* and *MEPE* are classic markers for the osteoid‐ or mineralizing‐osteocyte, stage (Bonewald, 2011; Kim & Adachi, 2019), which slightly increased over time under normoxic conditions but peaked after 21 days under hypoxic conditions, consistent with an increased rate of differentiation. In similar previous experiments (under normoxia), both genes were shown to peak relatively late in primary human osteoblast and Saos‐2 cultures during differentiation (Prideaux et al., 2014; Welldon et al., 2013). Most importantly, mRNA expression of the mature osteocyte markers *DMP1*, *SOST*, and *GJA1* (Paic et al., 2009; Poole et al., 2005; Toyosawa et al., 2001) increased significantly under both conditions but were relatively significantly increased under hypoxic conditions and plateaued by day 14, and at this time point reached a similar expression level as after 28 days under normoxic conditions. Overall, these findings are consistent with Saos‐2 cells under hypoxic conditions reaching an osteocyte‐like stage within 14 days.

Hypoxia has been shown to have profound effects on bone cell metabolism and osteoblast differentiation, although with sometimes contradictory findings (Usategui‐Martín et al., 2022). In primary rat calvarial osteoblasts, one study reported a hypoxia‐dependent decrease in mineralized nodule formation, associated with decreased alkaline phosphatase activity (Utting et al., 2006), while another found an increased expression of osteocyte markers and mineralization in MC3T3‐E1 cells, similar to our study (Hirao et al., 2007). Another study showed reduced *SOST* expression and increased Wnt/β‐catenin signaling in the rat UMR106.01 osteosarcoma and mouse MLO‐A5 osteoblastic cell lines, consistent with a pro‐anabolic effect of hypoxia, although effects on in vitro mineralization were not reported (Genetos et al., 2010). However, in our study, *SOST* expression was further increased under 1% O_2_, we suggest as a physiologic response to the increased mineralization and acquisition of a mature osteocyte‐like phenotype. The reasons for the observed differences between previous studies and our own are unclear. It is possible that the effect of hypoxia may either be cell type (e.g., species, skeletal origin, transformed *v*. primary) or methodology‐specific, as the above studies also employed different approaches to model hypoxia.

This modified Saos‐2 differentiation model has a number of applications in terms of improved feasibility and physiologic relevance. Notably, studies investigating human osteoblast (Saos‐2) on‐growth onto modified orthopedic implant surfaces, including those from our own group (Gulati et al., 2016; Qin et al., 2018), have typically utilized standard normoxic conditions, however the interface between an implant and the bone is usually avascular and therefore is hypoxic. In addition, Saos‐2 cells are a useful cell line model, with which to study the gene and protein expression regulation of *SOST*/sclerostin in a human context, for example in response to BMP stimulation (Yu et al., 2011), mechanical loading (Galea et al., 2013), and 1,25‐dihydroxyvitamin D_3_ treatment (Wijenayaka et al., 2015), and it is possible that examination under hypoxic conditions may increase the physiological relevance of such studies.

Another clear application of our model is in terms of studying infection by pathogens relevant to osteomyelitis, such as *S. aureus*. We have shown that osteocyte‐like Saos‐2 cells can internalize and harbor *S. aureus* (Gunn et al., 2021). Here, the two different differentiation conditions did not impact the bacterial invasion of the osteocyte‐like Saos‐2 cells, whether or not infection post‐Saos‐2 differentiation was also carried out under low oxygen. CFU recovery 24 h post‐infection was not significantly different between culture conditions. This is important, since we have previously shown that undifferentiated, osteoblast‐like cultures of Saos‐2 internalized significantly more (approximately 100‐fold) *S. aureus* than (28 days) differentiated osteocyte‐like cells. Therefore, accelerated differentiated cells can be used for infection assays as performed previously using day 28 differentiated cells (Gunn et al., 2021). *S. aureus* is known to adapt to hypoxic conditions (Balasubramanian et al., 2017; Lodge et al., 2017) and therefore it might be paramount to use hypoxic conditions for in vitro assays of *S. aureus* in osteomyelitis.

This study has a number of limitations. Firstly, the hypoxia model we chose utilized cell culture incubators with variable control of oxygen levels, however, these were uncontrolled during feeding, except that fresh medium was equilibrated by placing it in the incubator for 1–2 h prior to media exchange. It would therefore be expected that media oxygen levels would fluctuate during handling. Nevertheless, a profound effect on differentiation parameters was observed with our protocol. Secondly, we tracked osteocyte/osteoblast marker expression only at the mRNA level and did not confirm corresponding protein expression. This was due in part to the difficulties in performing immunohistochemistry on heavily mineralized cells and the relative paucity of validated antibodies to human osteocyte‐associated protein markers. For these reasons, it was necessary to examine a broad range of markers, as we performed here, to protect against the possibility that mRNA expression for a particular marker does not necessarily correspond to protein expression. As is evident here, there was a strong pattern of gene expression indicative of an osteocyte‐like phenotype, with arguably the most informative osteocyte markers, *SOST*, encoding sclerostin, *DMP1*, encoding dentin matrix protein‐1 and *GJA1*, encoding connexin 43, the major constituent of osteocyte gap junctions, being maximally expressed at the day 14 time point under low oxygen conditions. In addition, there was a strong pattern of concordance of gene expression with independent indicators of differentiation, including calcium phosphate mineral deposition (evidenced by Alizarin Red and Von Kossa staining), as well as alkaline phosphatase enzymic activity. Another study limitation is that we have only tested utility of our model with respect to studying osteocyte infection by *S. aureus*. We have not yet tested other measures of osteocyte functionality, such as responsiveness to PTH, although we predict that this function would be intact since we previously showed that Saos‐2 cells differentiated under normoxia responded to PTH by suppressing *SOST* mRNA expression throughout a 35‐day differentiation period (Prideaux et al., 2014). Finally, we have not investigated whether 1% O_2_ confers a similar effect on primary cell differentiation, and this would be of interest irrespective of the findings. However, the intent of this study was to examine this effect on a cell line model, for the purposes of establishing a reproducible model that is readily available and free of issues associated with primary cells, such as donor variability. Given that Saos‐2, to our knowledge, is the only available immortalized human cell line shown to be capable of differentiation to an osteocyte‐like stage, we consider this study to be an important advance.

In summary, Saos‐2 cells cultured under low oxygen exhibit accelerated in vitro mineralization and differentiation to a mature osteocyte‐like stage within 14 days. The resulting cells challenged with *S. aureus* showed similar infection dynamics to cells differentiated under standard conditions for 28 days. This significantly reduces the time and therefore costs required to generate mature human osteocyte‐like cultures, facilitating research into this important cell type. The model continues the advantage of using Saos‐2 cells due to their ease of availability over primary cells, and improved inter‐assay consistency independent of human donor variation. As a human cell line, the model may also provide findings of closer immediate clinical relevance than the extant non‐human in vitro models.

## AUTHOR CONTRIBUTIONS

ARZ performed most experiments, manuscript draft. YS performed pilot study. LBS, KR manuscript development. DY project design, manuscript development. GJA project conception, manuscript draft.

## FUNDING INFORMATION

This work was supported by funding from the National Health and Medical Research Council of Australia (NHMRC; Grant No. 2011042). AZ was supported by a University of Adelaide Faculty of Health and Medical Sciences Postgraduate Research Scholarship.

## CONFLICT OF INTEREST STATEMENT

None of the authors has any conflicts of interest, financial or otherwise, to disclose.

## ETHICS STATEMENT

This study required no research ethics approvals.
